# Robotic management of congenital urethra-vaginal fistula with transverse vaginal septum

**DOI:** 10.1590/S1677-5538.IBJU.2021.0421

**Published:** 2021-09-10

**Authors:** Shanti Laxmi Darga, Mallikarjuna Chiruvella, Taif Mohammed Bedigeri, Ghouse Syed Mohammed, Sarika Pandya, Bhavatej Enganti

**Affiliations:** 1 Asian Institute of Nephrology & Urology Department of Urology Hyderabad India Department of Urology, Asian Institute of Nephrology & Urology, Hyderabad, India

## Abstract

**Introduction:**

The transverse vaginal septum (TVS) with congenital urethra-vaginal fistula (CUVF) is a rare anomaly of the mullerian duct (1, 2). Incomplete channelling of the vaginal plate, or an abnormality in the fusion of the vaginal component of mullerian duct with the urogenital sinus results in TVS (1, 3, 4). High CUVF occurs due to the persistent communication between the urogenital sinus and utero-vaginal primordium at the tubercle sinus, whereas low CUVF is due to excessive apoptosis of the vaginal plate during channelling (5). The principles of management of CUVF with TVS include: 1) TVS resection, 2) Create a neovagina. We present a case of CUVF with TVS managed by robotic assistance.

**Material and methods:**

A 24-year-old female, married for 3 years, presented with cyclical hematuria since menarche, dyspareunia and primary infertility. Examination revealed blind ending vagina 4cm from the introitus. Magnetic resonance imaging revealed a fistulous communication between urethra and vagina, and TVS. Cystourethroscopy confirmed a proximal urethra-vaginal fistula. Urethroscopy guided puncture of the TVS was performed, tract dilated and a catheter was placed across it. Robotic assisted transvaginal approach was planned. Air docking of robot was performed. Traction on the catheter was given to identify the incised edges of the septum. Vaginal flaps were raised laterally, fistulous tract was excised. Proximal vagina mucosa was identified and vaginoplasty was performed.

**Result:**

Patient’s postoperative recovery was uneventful. Urethral catheter was removed after 5 days. She had normal voiding and menstruation. Vaginoscopy performed at 1st month follow-up, revealed an adequate vaginal lumen. Vaginal moulds were advised for 6 weeks during the night, following which she resumed her sexual activity. She conceived 6 months post-surgery, and delivered a child by caesarean section.

**Conclusion:**

We successfully managed this case by resection of septum, neovagina creation and thereby achieving normal menstruation and conception. The advantages of robotic approach were magnification, precision and manoeuvrability in a limited space, avoiding a vaginal release incision.
